# Comparison of Endoscopic Therapies for Small Rectal Neuroendocrine Tumors: Endoscopic Muscularis Superficialis Dissection Versus Endoscopic Submucosal Dissection

**DOI:** 10.1002/jgh3.70348

**Published:** 2026-02-07

**Authors:** Xiawen Shu, Xue Chen, Yirong Ding, Yun Yi, Lurao Li, Kun Li, Jiaoze Shi, Zhishan Chen, Xing Huang, Ying Chang

**Affiliations:** ^1^ The Second Clinical College of Wuhan University Wuhan China; ^2^ Department of Gastroenterology Zhongnan Hospital of Wuhan University Wuhan China

**Keywords:** endoscopic muscularis superficialis dissection, endoscopic submucosal dissection, endoscopic treatment, rectal neuroendocrine tumor

## Abstract

**Background and Aim:**

Rectal neuroendocrine tumors (rNETs) often exhibit submucosal tumor‐like growth. While endoscopic submucosal dissection (ESD) is widely used, it carries a risk of positive vertical margins, often necessitating repeated surveillance and imposing both financial and psychological burdens on patients. To address this limitation, we developed endoscopic muscularis superficialis dissection (EMSD), a technique involving controlled dissection into the superficial muscularis propria layer to improve complete resection rates. This study aimed to compare the therapeutic outcomes of EMSD and ESD for small rNETs.

**Methods:**

This retrospective study enrolled 82 patients (88 rNETs) undergoing ESD or EMSD between May 2019 and June 2025. Primary outcomes included complete resection rates, complication rates, and postoperative hospital stay.

**Results:**

The study analyzed 35 lesions treated with EMSD and 53 with ESD. Both groups had similar tumor characteristics. Compared to ESD, EMSD achieved significantly higher rates of both complete vertical margin resection (100% vs. 69.8%, *p* < 0.001) and R0 resection (100% vs. 67.9%, *p* < 0.001). However, there were no significant differences in procedure time (47.0 ± 17.0 min vs. 40.0 ± 9.5 min; *p* = 0.070) and postoperative hospital stay (4.0 ± 1.5 days vs. 4.0 ± 1.0 days; *p* = 0.676). Postoperative bleeding occurred in 1 EMSD patient (2.9%), which was managed endoscopically. No other bleeding or perforation cases occurred.

**Conclusions:**

Compared with ESD, EMSD achieved superior performance in the resection of rNENs ≤ 10 mm in diameter regardless of submucosal invasion depth.

## 1 Introduction

The incidence of neuroendocrine tumors (NETs) has risen steadily over the past decade [1]. The rectum is the second most common site after the appendix [2], accounting for 15%–55% of all gastrointestinal NETs [3, 4]. Rectal neuroendocrine tumors (rNETs) possess malignant potential [5]. Tumor size is one of the key factors in evaluating the malignant potential of rNETs [6]. Although small rNETs (≤ 10 mm) are generally indolent, they still carry a small but clinically relevant risk of metastasis [7]. Current guidelines recommend endoscopic resection for these lesions [8]. However, achieving R0 resection, particularly at the vertical margin, remains a challenge.

The 2023 European Neuroendocrine Tumor Society (ENETS) guidelines for colorectal NETs recommend endoscopic techniques such as endoscopic mucosal resection (EMR), modified EMR (mEMR), endoscopic submucosal dissection (ESD), and endoscopic full‐thickness resection (EFTR) for lesions ≤ 10 mm [8]. Standard EMR has a modest R0 resection rate (~50%), prompting a preference for ESD (> 80% R0 resection rate) in modern endoscopic practice [8, 9, 10]. Although recent mEMR techniques have demonstrated enhanced R0 resection rates compared with ESD, they remain suboptimal and are indicated primarily for tumors with superficial submucosal infiltration (SM1) [7, 11]. For small submucosal‐confined rNETs, EFTR may lead to overtreatment.

ESD is recommended for rNETs measuring ≤ 10 mm and even for those measuring 1–2 cm [8]. However, since gastrointestinal NETs often present as submucosal tumors (SMTs) [12], ESD may still result in positive vertical margins [13]. Incomplete endoscopic resection can induce fibrosis, potentially compromising the efficacy of subsequent endoscopic interventions [14, 15]. Moreover, residual tumor tissue persists in 17%–43% of cases following R1 resection [8, 16], which itself is an independent predictor of recurrence [17]. Additionally, studies indicate that small rNETs ≤ 10 mm retain a 3% risk of distant metastasis [18], necessitating repeated endoscopic surveillance and supplementary radiological examinations.

Endoscopic submucosal excavation (ESE), an advanced variant of ESD, facilitates deeper dissection into the muscularis propria layer [19]. To achieve greater anatomical precision for rectal dissection given its distinct bilayered muscularis propria [20], we have employed and defined the term “endoscopic muscularis superficialis dissection (EMSD)” in this study. EMSD specifically denotes a controlled dissection within the superficial (circular) muscular layer for resecting small rNETs. The efficacy of EMSD for resecting rNETs, however, has not been thoroughly evaluated.

In summary, we hypothesize that EMSD is a better choice for rNETs ≤ 10 mm in diameter. Here we report our experience and aim to evaluate and compare the therapeutic efficacy and safety.

## 2 Materials and Methods

### 2.1 Patients

We retrospectively analyzed 82 patients with rNETs who underwent ESD or EMSD procedures at Zhongnan Hospital of Wuhan University (May 2019 to June 2025). The inclusion criteria were as follows: lesions ≤ 10 mm in diameter with a hemispherical and smooth mucosal surface observed during colonoscopy, which were pathologically confirmed as rNETs after resection. The exclusion criteria included: muscularis propria invasion by endoscopic ultrasound (EUS); lymph node or distant metastasis detected on computed tomography (CT); coagulation abnormalities; severe comorbidities; and concurrent malignancies. The study received approval from the institutional review board of our hospital (approval number: 2024321K).

**FIGURE 1:**
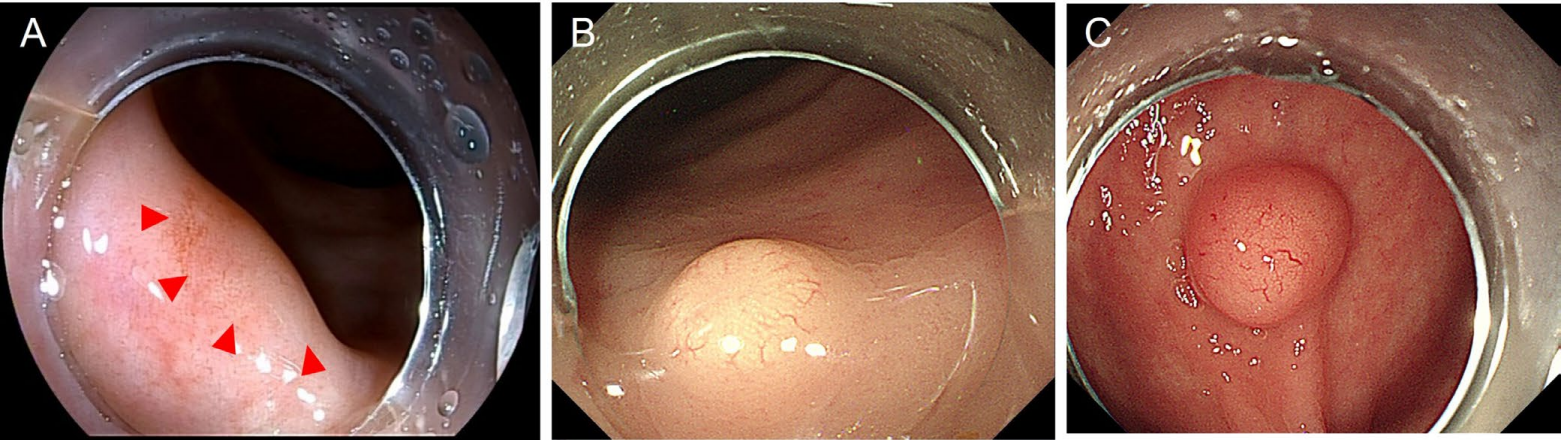
Endoscopic classification of rNETs morphology. (A) Type 1 (Flat): Lesions with a completely flat or minimally elevated profile (< 2 mm, indicated by the red triangle). (B) Type 2 (Slightly elevated): Lesions with low, broad‐based elevation (2–4 mm), and central depression or biopsy marks. (C) Type 3 (Distinctly elevated): Lesions with prominent, polypoid or hemispherical elevation (> 4 mm) and smooth mucosal surface.

The choice between EMSD and ESD was mainly based on lesion morphology and EUS‐assessed invasion depth. Lesions were categorized into three types (Figure 1): Type 1 (Flat): completely flat or minimally elevated (< 2 mm); Type 2 (Slightly elevated): low, broad‐based elevation (2–4 mm), often with central depression or biopsy marks; Type 3 (Distinctly elevated): prominent, polypoid or hemispherical elevation (> 4 mm) with a smooth surface. Due to their higher risk of deep submucosal infiltration [21], Type 1 and 2 lesions were preferentially assigned to EMSD to ensure a sufficient deep margin. Conversely, Type 3 lesions, which typically grow upward with lower invasion risk, were often treated with ESD. EMSD was also indicated when EUS suggested deep submucosal invasion (exceeding two‐thirds of the submucosa). The final decision integrated these criteria with individual lesion characteristics (e.g., lifting sign after injection, presence of fibrosis) and clinical judgment to optimize the chance of R0 resection.

### 2.2 ESD, EMSD Procedures

**FIGURE 2:**
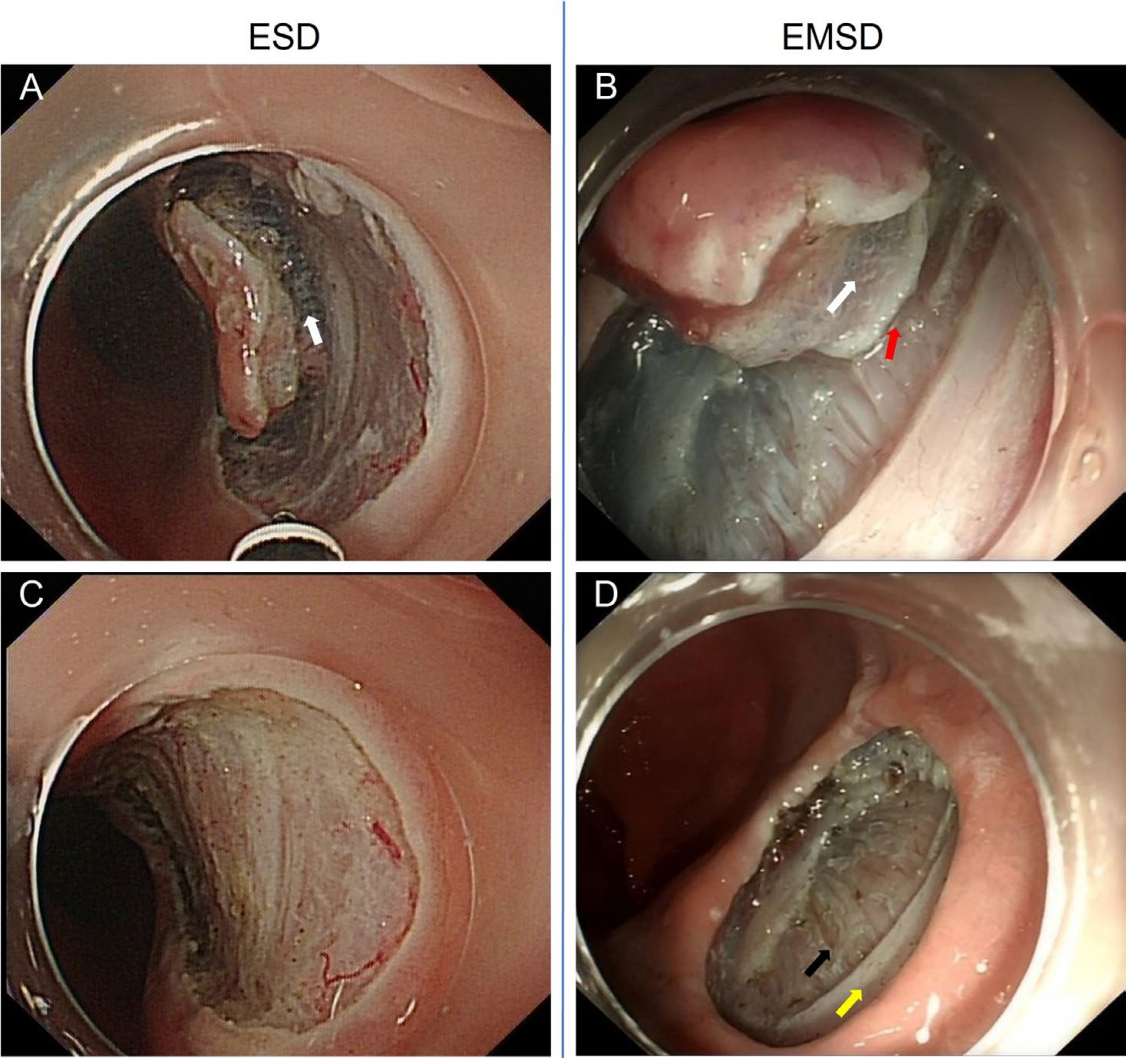
Dissection planes and wound surfaces of ESD and EMSD. (A) ESD is performed within the submucosal layer (white arrow). (B) EMSD dissection proceeds between the superficial and deep layers of the muscularis propria (red arrow). (C) Post‐ESD wound surface: The muscularis propria is intact. (D) Post‐EMSD wound surface: the superficial circular muscle is incised (yellow arrow), while the deep longitudinal muscle remains intact (black arrow).

Both ESD and EMSD procedures began with consistent initial steps: electrocautery marking approximately 5 mm outside the tumor margin, followed by submucosal injection of a mixture containing normal saline, indigo carmine, and sodium hyaluronate to lift the lesion. After resection, any visible bleeding sites were coagulated, and all wounds subsequently underwent prophylactic closure with clips. The core distinction between the two techniques lies in the depth and endpoint of submucosal dissection (Figure 2).

**FIGURE 3:**
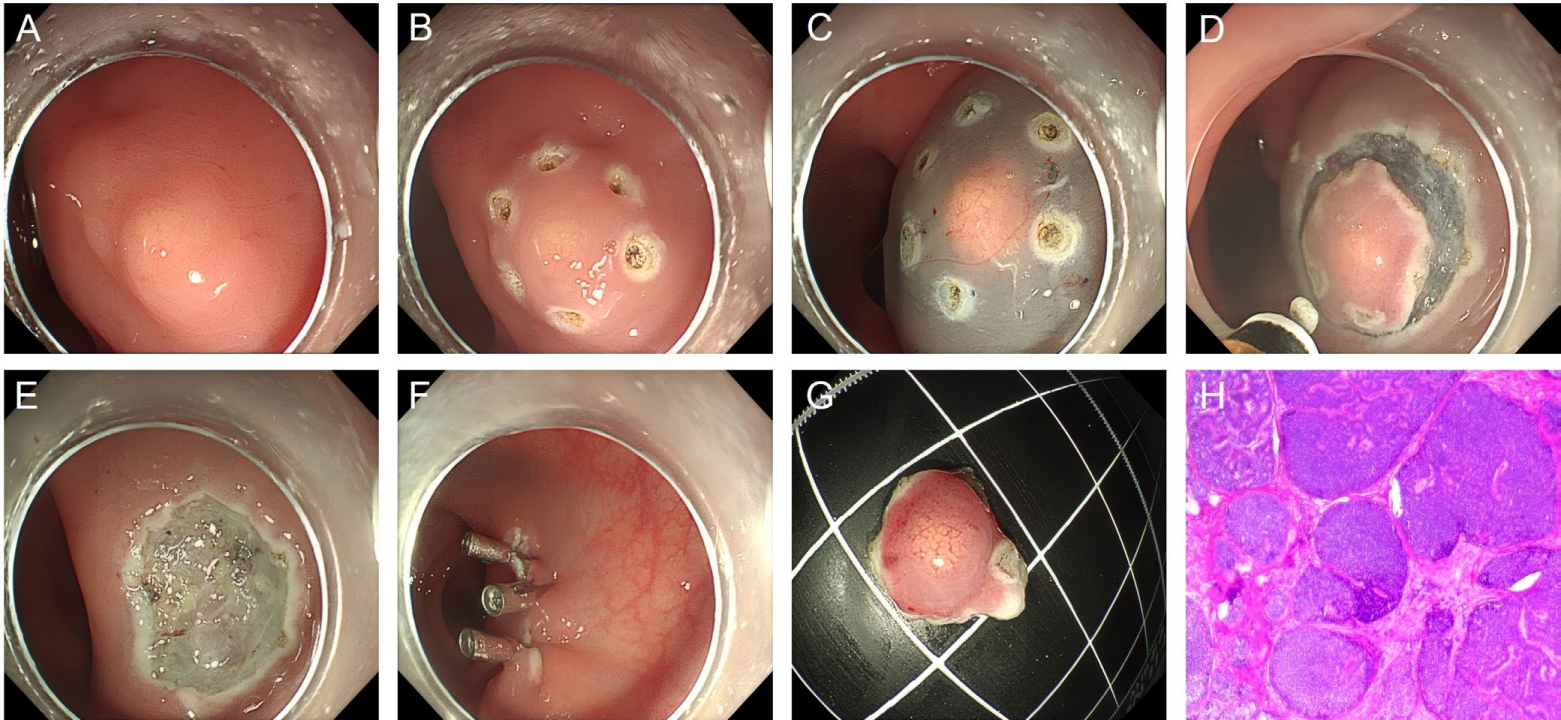
Endoscopic submucosal dissection (ESD) procedure. (A) Approximately 6 mm rectal mucosal protrusion; (B) circumferential marking; (C) Submucosal injection; (D) Stepwise dissection along the submucosal layer; (E) Post‐ESD; (F) Clamp the wound; (G) En bloc resection specimen; (H) Pathology confirmed a rNET (G1) with clear lateral and deep margins (H&E staining).

ESD: Following submucosal injection, a circumferential mucosal incision was made outside the lesion margin. Subsequent dissection was confined to the submucosal layer, maintaining the plane between the submucosa and the muscularis propria. The procedural endpoint was characterized by en bloc resection of the lesion at its base, with the underlying muscularis propria remaining intact and uninjured (Figure 3).

**FIGURE 4:**
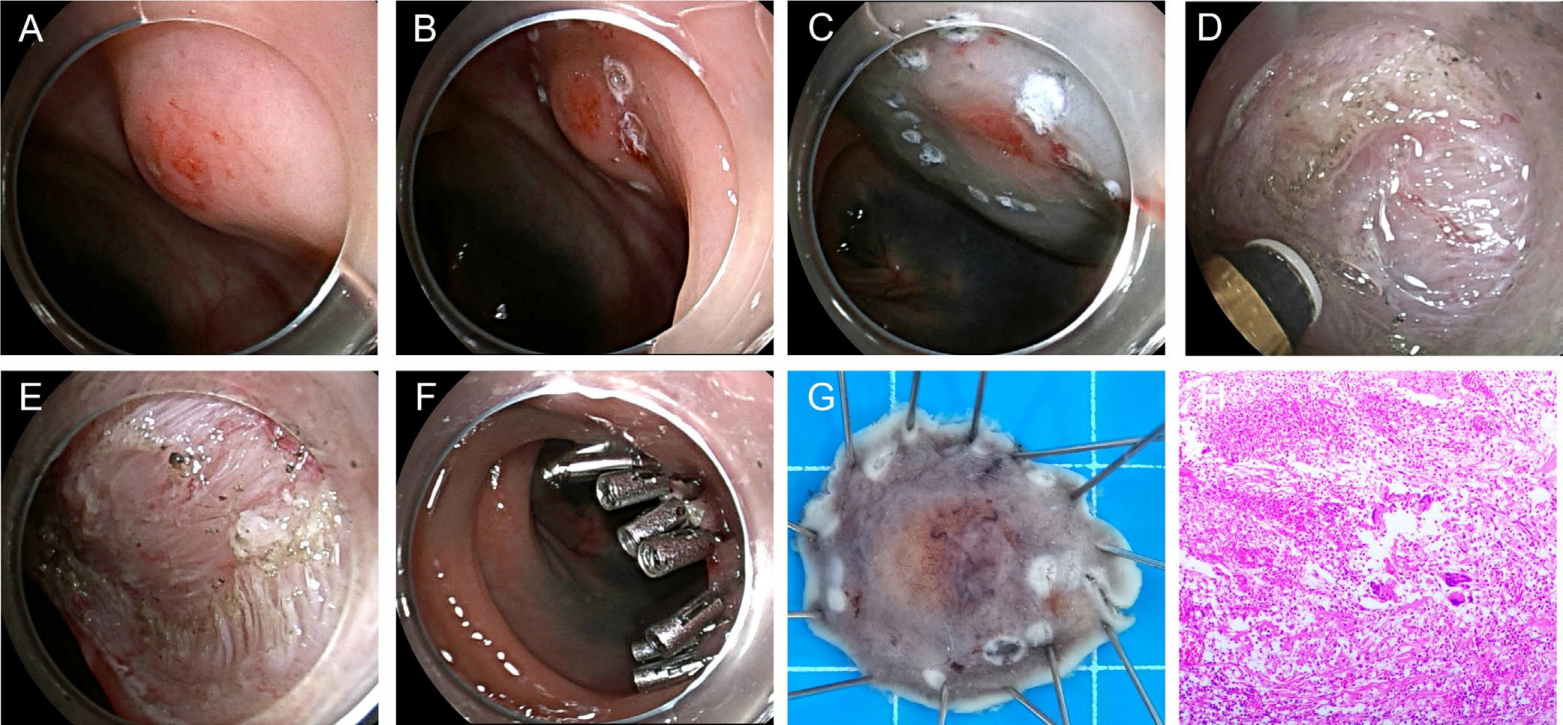
Endoscopic muscularis superficialis dissection (EMSD) procedure. (A) Approximately 10 mm rectal mucosal protrusion; (B) circumferential marking; (C) submucosal injection; (D) stepwise dissection and partial resection of the superficial muscle layer; (E) post‐EMSD; (F) Clamp the wound; (G) En bloc resection specimen; (H) pathology confirmed a rNET (G1) with clear lateral and deep margins (H&E staining).

EMSD: After the mucosal incision, we performed a controlled, active dissection within the superficial muscularis propria (circular muscle), carefully preserving the underlying deep longitudinal muscle layer and the serosa (Figure 4). Consequently, the procedural endpoint was the creation of a superficial, controlled defect in the muscularis propria following lesion removal.

It should be noted that EMSD shares conceptual similarities with endoscopic intermuscular dissection (EID) in utilizing the intermuscular plane. However, they differ fundamentally in indications, technical starting point, and extent of dissection. EID is primarily indicated for tumors originating from or invading the muscularis propria [21, 22]. The procedure involves directly identifying and entering the intermuscular plane from the submucosal layer, followed by relatively extensive dissection to completely enucleate the intramural lesion. In contrast, EMSD is designed for rNETs with deep submucosal infiltration. Its defining maneuver is the controlled incision of superficial circular muscle fibers at the tumor base to access the intermuscular plane for a superficial and confined dissection. EMSD thus represents a more targeted and localized technique that prioritizes preservation of the deep muscular layer to secure a radical vertical margin while theoretically reducing the perforation risk compared to EID.

## 3 Evaluation of Histological and Complications

A positive resection margin was defined as pathological evidence of tumor involvement at either vertical or lateral margin, or tumor proximity to the vertical or horizontal margin [23, 24]. R0 resection required both horizontal and vertical margins to be negative, whereas R1 resection was classified when either the vertical or lateral margin was positive. All cases underwent histopathological assessment of tumor type, lymphovascular invasion (LVI), and perineural invasion (PNI). Tumor grading (G1/G2/G3) followed the 2019 WHO classification [25].

Hemorrhage and perforation were mainly postoperative complications. Hemorrhage was defined as hematochezia requiring endoscopic hemostasis within 2 weeks postoperatively. Perforation was defined as intraoperatively visualized full‐thickness defect or radiologically (abdominal plain radiography or CT) confirmed free air.

## 4 Salvage Therapy and Follow‐Up

Additional treatments such as endoscopic resection and transanal minimally invasive surgery were recommended for patients with LVI, muscularis propria invasion, R1 resection, and G2/G3 pathological grading [8]. These patients also received more intensive endoscopic surveillance in addition to routine follow‐up care. Given the reported 22.7% incidence of lymphatic metastasis in multifocal rNETs ≤ 10 mm in diameter [26], salvage procedures were implemented for cases with R1 resection margins.

Patients underwent standardized endoscopic surveillance at 3, 6, and 12 months postoperatively, and then took annual examinations (abdominopelvic CT or MRI, endoscopy) to monitor for recurrence and metastasis.

## 5 Statistical Analysis

Continuous variables are expressed as the mean ± standard deviation and range, while categorical variables are reported as counts and percentages. Group differences were assessed using the chi‐square test for categorical data and Student's *t*‐test or the Mann–Whitney *U* test for continuous data, as appropriate. Statistical significance was set at *p* < 0.05. All analyses were performed using SPSS version 27.0 software.

## 6 Results

### 6.1 Characteristics of the Patients and Tumors

In total, we collected 88 cases of rNETs (82 patients). Patient and tumor characteristics are summarized in Table 1. Both groups had similar age distribution (EMSD 50.26 ± 12.10, ESD 52.08 ± 11.85; *p* = 0.486). The mean endoscopic estimated tumor diameters (EMSD 0.7 ± 0.25 cm, ESD 0.6 ± 0.15 cm; *p* = 0.288) and anal margin distance (EMSD 6.96 ± 2.41 cm, ESD 6.36 ± 2.33 cm; *p* = 0.526) were also similar. Tumors were mainly located in the distal rectum. While sex distribution differed significantly between groups (*p* = 0.014), the effect size was small (φ = 0.26, Cohen's criteria) and the limited sample size suggests minimal clinical relevance. Larger studies are warranted to further confirm this significance.

**TABLE 1:** Patient and tumor characteristics for the EMSD and ESD groups.

Variables	EMSD (*n* = 35)	ESD (*n* = 53)	*p* value
Age (years)			0.486^c^
Mean ± SD	50.26 ± 12.10	52.08 ± 11.85	
Median (range)	51 (32–72)	52 (23–75)	
Gender, *n* (%)			0.014^d^
Male	16 (45.7)	38 (71.7)	
Female	19 (54.3)	15 (28.3)	
Previous mechanical stimulation history, *n* (%)			0.167^e^
Yes	6 (17.1)	3 (5.7)	
No	29 (82.9)	50 (94.3)	
Anal margin distance (cm)			0.526^c^
Mean ± SD	6.69 ± 2.41	6.36 ± 2.33	
Median (min–max)	6 (1–12)	6 (1–13)	
Endoscopic estimated tumor size (cm)			0.288^f^
Median ± semi‐IQR^a^	0.7 ± 0.25	0.6 ± 0.15	
Median (range)	0.7 (0.2–1.0)	0.6 (0.2–1.0)	
Lesion morphology, *n* (%)			0.032^d^
Type 1 and 2	24 (68.6)	24 (45.3)	
Type 3	11 (31.4)	29 (54.7)	
Deep submucosal invasion on EUS, *n* (%)			< 0.001^d^
Yes (depth ≥ 2/3 submucosa)	28 (80.0)	0 (0)	
No	7 (20.0)	53 (100)	
Tumor level (distance from AV, cm)^b^			0.797^d^
Upper rectum	6 (17.1)	8 (15.9)	
Lower rectum	29 (82.9)	45 (84.1)	
LVI, *n* (%)			0.646^e^
Yes	1 (2.9)	4 (7.5)	
No	34 (97.1)	49 (92.5)	
PNI, *n* (%)			1.000^g^
Yes	0 (0)	1 (1.9)	
No	35 (100)	52 (98.1)	
Histologic grade			0.445^d^
G1	26 (74.3)	43 (81.1)	
G2	9 (25.7)	10 (18.9)	
Infiltration depth			0.556^g^
Mucosa	2 (5.7)	1 (1.9)	
Submucosa	33 (94.3)	52 (98.1)	

Abbreviations: AV: anal verge, EMSD: endoscopic muscularis superficialis dissection, ESD: endoscopic submucosal dissection, LVI: lymphovascular invasion, SD: standard deviation, PNI: perineural invasion.

^a^ For both groups (non‐normally distributed), data is expressed as median ± half the interquartile range (IQR/2).

^b^ Based on the distance from the tumor to the anal margin, a distance of ≤ 8 cm is considered the lower rectum, while distances > 8 cm are considered the upper rectum.

^c^ Independent samples *t*‐test.

^d^ Pearson *χ* ^2^ test.

^e^ Correction for Continuity *χ* ^2^ test.

^f^ Mann–Whitney *U* test.

^g^ Fisher exact test.

As per our clinical decision protocol, lesions with high‐risk morphology (Type 1 and 2) and deep submucosal invasion (depth ≥ 2/3 on EUS) were more prevalent in the EMSD group (68.6% vs. 45.3%, *p* = 0.032; and 80% vs. 0%, *p* < 0.001, respectively). Histopathological analysis revealed predominantly well‐differentiated (G1) tumors (74.3% vs. 81.1%, *p* = 0.445), with most confined to the submucosa (94.3% vs. 98.1%, *p* = 0.556) and no poorly differentiated (G3) cases identified.

### 6.2 Endoscopic Resection Results

Table 2 summarized the results of endoscopic treatment in both groups. Each group demonstrated a 100% rate of endoscopic complete resection. However, the EMSD group achieved a higher complete vertical margin resection rate (100% vs. 69.8%, *p* < 0.001). The R0 resection rate was also significantly higher in the EMSD group (100% vs. 67.9%, *p* < 0.001). Meanwhile, the EMSD group showed performance comparable to ESD in terms of procedure time (47.0 ± 17.0 min vs. 40.0 ± 9.5 min, *p* = 0.070) and postoperative hospital stay (4.0 ± 1.5 days vs. 4.0 ± 1.0 days, *p* = 0.676).

**TABLE 2:** Endoscopic resection and pathological results.

Variables	EMSD (*n* = 35)	ESD (*n* = 53)	*p* value
Endoscopic complete resection, *n* (%)			NA
Negative	35 (100)	53 (100)	
Positive	0 (0.0)	0 (0.0)	
Histologic outcome			
Vertical margin, *n* (%)			< 0.001^a^
Negative	35 (100)	37 (69.8)	
Positive	0 (0)	16 (30.2)	
Lateral margin, *n* (%)			1.000^c^
Negative	35 (100)	52 (98.1)	
Positive	0 (0)	1 (1.9)	
Resection status, *n* (%)			< 0.001^a^
R0 resection	35 (100)	36 (67.9)	
R1 resection	0 (0)	17 (32.1)	
Procedure time (minutes)			0.070^d^
Median ± semi‐IQR	47.0 ± 17.0	40.0 ± 9.5	
Median (range)	47 (21–96)	40 (17–82)	
Postoperative hospital stay			0.676^d^
Median ± semi‐IQR	4.0 ± 1.5	4.0 ± 1.0	
Median (range)	4 (2–15)	4 (2–12)	
Adverse event, *n* (%)			0.398^c^
Yes	1 (2.9)	0 (0)	
No	34 (97.1)	53 (100)	
Rescue surgery, *n* (%)			0.819^b^
Yes	2 (5.7)	5 (9.4)	
No	33 (94.3)	48 (90.6)	
Recurrence, *n* (%)			NA
Yes	0 (0)	0 (0)	
No	35 (100)	53 (100)	

Abbreviations: EMSD: endoscopic muscularis superficialis dissection, ESD: endoscopic submucosal dissection, NA: not applicable.

^a^ Pearson *χ* ^2^ test.

^b^ Correction for continuity *χ* ^2^ test.

^c^ Fisher exact test.

^d^ Mann–Whitney *U* test.

### 6.3 Relationship Between Clinicopathologic Factors and Resection Status

To identify factors influencing R0/R1 resection, we analyzed endoscopic tumor size, tumor location, histopathological grade, EMR or previous biopsy history, infiltration depth, endoscopist's expertise (see table 3 notes for definitions [27]) as shown in Table 3. The data provided no conclusive evidence for an association between the two groups (all *p* > 0.05), suggesting that the choice of endoscopic technique may be the primary determinant.

**TABLE 3:** Relationship between clinicopathologic factors and resection margin.

Variables	R0 resection (*n* = 71)	R1 resection (*n* = 17)	*Z*/*χ* ^2^ value	*p* value
Tumor size (cm)			0.422	0.673^e^
Median ± semi‐IQR	0.6 ± 0.15	0.6 ± 0.17		
Median (range)	0.6 (0.2–1.0)	0.6 (0.3–1.0)		
Anal margin distance (cm)			0.674	0.501^e^
Mean ± SD	6.0 ± 1.50^a^	6.1 ± 1.81		
Median (min–max)	6.0 (1–13)	6.0 (3–10)		
Pathological grade, *n* (%)			/	1.000^d^
G1	56 (78.9)	13 (76.5)		
G2	15 (21.1)	4 (23.5)		
Infiltration depth			/	1.000^d^
Mucosa	3 (4.2)	0 (0)		
Submucosa	68 (95.8)	17 (100)		
Previous biopsy, *n* (%)			/	1.000^d^
Yes	8 (11.3)	1 (5.9)		
No	63 (88.7)	16 (94.1)		
Endoscopist's expertise^b^			0.944	0.331^c^
Senior endoscopists	30 (42.3)	6 (29.4)		
Junior endoscopists	41 (57.7)	11 (70.6)		

Abbreviation: SD: standard deviation.

^a^ For the R0 resection group (non‐normally distributed), data is expressed as median ± IQR/2.

^b^ The operator experience was stratified by annual procedural volume. Senior endoscopists: ≥ 80 combined ESD/EMSD cases/year. Junior endoscopists: < 80 total ESD/EMSD cases annually. All of them have been engaged in endoscopic work for more than 2 years.

^c^ Pearson *χ* ^2^ test.

^d^ Fisher exact test.

^e^ Mann–Whitney *U* test.

### 6.4 Complications

One bleeding case was observed in the EMSD group and was successfully controlled by endoscopic hemostasis, while none occurred in the ESD group. No patients experienced perforation events, with no difference in complication rates between the two groups.

### 6.5 Follow‐Up

Follow‐up data were available for 27 patients in the EMSD group and 40 in the ESD group. 10 patients had not yet reached the 3‐month follow‐up timepoint (EMSD 8, ESD 2). Median follow‐up duration was 12 months (range 1–42 months). In the EMSD group, one patient underwent salvage surgery with follow‐up at 1 month, while five patients in the ESD group received salvage procedures with follow‐up between 3 and 5 months. Recurrence did not occur in any patients. The clinicopathological characteristics of these salvage cases are presented in Table S1.

## 7 Discussion

RNETs are typically asymptomatic and incidentally detected by colonoscopy [28]. Lesions ≤ 10 mm show low malignant potential and are amenable by endoscopic resection [29], though there are not clear recommendations for endoscopic approach selection. Regardless of approach, achieving R0 resection is critical to avoid repeat procedures, additional surgeries, and patient distress [30].

According to the guidelines and previous studies [8, 11, 31], mEMR techniques such as ligation‐assisted EMR (EMR‐L) and cap‐assisted EMR (EMR‐C) are recommended as first‐line treatments for most small submucosal‐confined rNETs, owing to their technical simplicity, rapid operation, high R0 resection rate (~95%), and low complication risk. Although a positive margin may be managed with a watch‐and‐wait strategy, the psychological burden on patients drives the pursuit of complete resection. This is particularly critical for lesions with flat morphology, deeper submucosal invasion, or fibrosis, where mEMR carries an increased risk of R1 resection. Consequently, ESD is frequently preferred for such challenging lesions, as it enables en bloc resection and precise dissection around fibrotic tissue. Nevertheless, ESD is limited by its restricted vertical resection depth, with a vertical negative margin rate of approximately 85%. Recent studies have shown that EFTR can achieve a 100% R0 resection rate for lesions measuring ≤ 10 mm and 10–20 mm [23, 32], with operative time and postoperative adverse event rates comparable to those of ESD. Nevertheless, EFTR is more invasive and may represent overtreatment for rNETs ≤ 10 mm. Therefore, is there a compromise technique that can overcome the depth limitations of ESD while being less invasive than EFTR? We believe that EMSD precisely fills this gap.

Our study is the first one to compare EMSD and ESD for the treatment of rNETs ≤ 10 mm. EMSD demonstrated significantly higher rates of histologically complete vertical resection and R0 resection compared with ESD, achieving a 100% complete resection rate. Importantly, no significant differences were observed in operation duration, hospitalization, and complications. These findings carry important clinical implications. By overcoming the vertical depth limitation of ESD while maintaining a less invasive profile than EFTR, EMSD offers a valuable therapeutic option for challenging cases, particularly those with flat morphology, deep infiltration, or fibrosis. Furthermore, as demonstrated in our salvage cases (Table S1), EMSD serves as an effective rescue procedure following R1 resection, as its feasibility remains unaffected by pre‐existing submucosal defects or fibrosis. Additionally, our analysis of factors influencing R0 resection confirms that endoscopic technique choice is a key determinant, emphasizing its role in preoperative planning. Notably, even small rNETs may present adverse pathological features, underscoring the need for definitive R0 resection. In this context, EMSD demonstrates clear utility as an initial intervention for lesions with aggressive histology, potentially reducing the need for additional salvage procedures.

Our study has several limitations. First, its retrospective, single‐center design with a limited sample size and non‐randomized treatment allocation may introduce selection bias and limit generalizability. Furthermore, the absence of a direct comparison with guideline‐recommended mEMR techniques limits conclusions regarding the relative position of EMSD in the treatment algorithm. Second, although operator experience was not a significant factor in our cohort, the technical demands of EMSD suggest a learning curve. Our results originate from a high‐volume tertiary center and therefore require validation in broader practice settings. Third, the follow‐up period is too short to assess long‐term oncologic outcomes. Finally, the R0 rate in our ESD group was lower than in some previous reports, which may reflect our stringent histologic criteria for margin assessment and the modest sample size. These limitations underscore the need for future prospective, multicenter, randomized trials with larger cohorts and extended follow‐up to validate our findings and to directly compare EMSD with established methods such as mEMR for managing small rNETs.

In conclusion, EMSD is a safe and effective technique for small rNETs, achieving superior vertical margin clearance compared with ESD. It overcomes the depth limitation of ESD and complements mEMR and EFTR within a stratified treatment strategy. Despite its technical complexity, the clinical benefit it provides warrants its consideration in clinical practice.

## Funding

This work was supported by the National Natural Science Foundation of China (grant number 82172983).

## Ethics Statement

The study received approval from the institutional review board of Wuhan University Zhongnan Hospital (approval number: 2024321K).

## Conflicts of Interest

The authors declare no conflicts of interest.

## Supporting information


**Table S1:** Clinical‐pathological information sheet for salvage surgery patients.

## Data Availability

The data that support the findings of this study are available on request from the corresponding author. The data are not publicly available due to privacy or ethical restrictions.
