# Supplementary material for: Comparison of Endoscopic Therapies for Small Rectal Neuroendocrine Tumors: Endoscopic Muscularis Superficialis Dissection Versus Endoscopic Submucosal Dissection
Source: JGH Open. 2026 Feb 7;10(2):e70348. doi: 10.1002/jgh3.70348 (PMC12881991; doi:10.1002/jgh3.70348)
Supplement: Supplementary file 1 — Table S1: Clinical‐pathological information sheet for salvage surgery patients. [file JGH3-10-e70348-s001.docx]

**Supplementary Table. 1 Clinical-pathological information sheet for salvage surgery patients**

| Case | Group | Age (year) | Gender | Anal margin distance (cm) | Tumor size (cm) | Infiltration depth | Grade | LVI | PNI | Resection status | Salvage therapy |
| --- | --- | --- | --- | --- | --- | --- | --- | --- | --- | --- | --- |
| 1 | ESD | 61 | male | 13 | 0.8 | Submucosa | G1 | (+) | (-) | R0 | Surgical operation |
| 2 |  | 65 | female | 8 | 0.5 | Submucosa | G2 | (-) | (-) | R1 | EFTR |
| 3^e^ |  | 51 | male | 6 | 0.5 | Submucosa | G1 | (-) | (-) | R1 | EMSD |
| 4 |  | 45 | male | 8 | 0.8 | Submucosa | G2 | (+) | (-) | R1 | Surgical operation |
| 5 |  | 45 | male | 6 | 0.4 | Submucosa | G2 | (+) | (-) | R0 | Surgical operation |
| 6 | EMSD | 36 | male | 8 | 0.8 | Submucosa | G1 | (+) | (-) | R0 | Surgical operation |

EMSD endoscopic muscularis superficialis dissection, EFTR endoscopic full-thickness resection, LVI lymphovascular invasion, PNI perineural invasion

e Case 3 was a patient with multiple NETs, in whom EMSD was performed as salvage therapy for an R1-resected lesion.
